# Minocycline and doxycycline therapy in community patients with rheumatoid arthritis: prescribing patterns, patient-level determinants of use, and patient-reported side effects

**DOI:** 10.1186/ar3491

**Published:** 2011-10-18

**Authors:** Christopher J Smith, Harlan Sayles, Ted R Mikuls, Kaleb Michaud

**Affiliations:** 1 Department of Internal Medicine, University of Nebraska Medical Center, 986270 Nebraska Medical Center, Omaha, NE 68198-6270, USA; 2 National Data Bank for Rheumatic Diseases, 1035 North Emporia, Suite 288, Wichita, KS 67214, USA

## Abstract

**Introduction:**

Minocycline and doxycycline are safe and moderately effective disease-modifying anti-rheumatic drugs (DMARDs) in the treatment of early, DMARD-naïve rheumatoid arthritis (RA), although little is known about their use outside clinical trials. We characterize the use of minocycline and doxycycline in community-dwelling RA patients by examining associated prescribing patterns, patient-level determinants of use, and side-effect profiles.

**Methods:**

We studied 15,716 patients with RA observed between 1998 and 2009 while participating in a long-term US observational study.

**Results:**

Minocycline or doxycycline was prescribed by 18% of rheumatologists (interquartile range one to two patients per physician) to 9% of RA patients. Significant differences between minocycline-treated and doxycycline-treated patients and nontreated patients included age (58.4 years vs. 59.8 years), RA duration (14.8 years vs. 13.7 years), Caucasian race (93.7% vs. 89.7%), lifetime DMARDs and biologics (3.3 vs. 2.5), prednisone use (40.1% vs. 35.3%), and Medical Outcomes Study 36-Item Short Form Survey physical component summary score (35.0 vs. 36.4). In multivariable Cox regression, patients initiating minocycline or doxycycline had increased disease activity, more comorbidities, and a greater number of prior nonbiologic DMARDs. Side effects were reported by 17.8% of minocycline users and 11.8% of doxycycline users. Skin complaints accounted for 54% of minocycline patient-reported side effects. The most commonly effected organ systems for doxycycline were gastrointestinal (35.4%) and skin (33.7%). Approximately 75% of side effects were of mild or moderate severity.

**Conclusions:**

Rheumatologists have not embraced minocycline or doxycycline as primary treatment options for RA and reserve their use primarily in patients with long-standing, refractory disease. These drugs are generally well tolerated, with skin complaints, nausea, and dizziness being the most common patient-reported side effects.

## Introduction

Minocycline and doxycycline are semi-synthetic tetracycline antibiotics with anti-inflammatory properties that are used to treat multiple inflammatory diseases, including rheumatoid arthritis (RA) [1,2]. Tetracyclines exhibit multiple anti-inflammatory properties, including the inhibition of T-cell activation and chemotaxis, the downregulation of proinflammatory cytokines, including TNFα and IL-1β [1-3], and the inhibition of matrix metalloproteinases [4-6].

Minocycline has proven to be a very safe and moderately effective disease-modifying antirheumatic drug (DMARD) in the treatment of RA, but its efficacy appears to vary greatly depending on the patient population in which it is used. Although an initial open-label study using minocycline in treatment-resistant RA was encouraging [7], two subsequent double-blind, placebo-controlled studies from the 1990s found only modest, although statistically significant, clinical improvement. The participants in these latter two trials had long-standing, DMARD-refractory disease [8,9]. In contrast, more recent trials examining minocycline in DMARD-naïve, early RA yielded more impressive results. In separate studies, minocycline showed superior efficacy and similar tolerability to placebo [10,11] and hydroxychloroquine [12].

Reports of doxycycline in the treatment of RA have also been inconsistent, with two studies showing no treatment benefit in patients with established disease [13,14] while a more recent study of patients with early disease showed significant efficacy compared with placebo when used in combination with methotrexate [15]. The benefit of minocycline and doxycycline was confirmed in a recent meta-analysis that found clinically significant improvement in disease activity with no increased risk for adverse events, although the authors note that the overall number and quality of clinical trials was low [16].

Although these studies indicate that minocycline and doxycycline represent important options in the treatment of RA, particularly among patients with recent-onset seropositive disease, little is known about how these medications are utilized outside the context of clinical trials. We sought to characterize the use of minocycline and doxycycline in a large community cohort of RA patients, by examining associated prescribing patterns, patient-level determinants of use, and the frequency and severity of patient-reported side effects.

## Materials and methods

### Study participants

Patients were diagnosed with RA by their physicians and were participants in the National Data Bank for Rheumatic Diseases (NDB) longitudinal observational study of RA outcomes. NDB participants are recruited from a large network of rheumatologists and fill out detailed, semi-annual questionnaires, which have been previously described [17-19]. Study participants were enrolled in the NDB from 1998 through 2009 and were not part of a drug safety registry as these patients may have more severe disease and may not be representative of the general RA population. Patients were categorized based on exposure to minocycline and/or doxycycline and the timing of drug initiation (prior to or after NDB enrollment).

### Study variables

The sociodemographic information analyzed includes age, male sex, Caucasian non-Hispanic race, education in years, total household income in US dollars, semi-annual direct medical expenses in US dollars [20], semi-annual expenses for all medications in US dollars, and insurance profile (private, HMO, Medicare, PPO, Medicaid, or no insurance). Disease characteristics include: duration of RA; rheumatic disease comorbidity index (range 0 to 9) [21]; cumulative use of biologic and nonbiologic DMARDs; concomitant use of methotrexate or prednisone; Medical Outcomes Study 36-Item Short Form Survey physical component summary score and mental component summary score [22]; Health Assessment Questionnaire disability index [23]; pain visual analog scale; patient global disease severity visual analog scale; and Patient Activity Scale [24,25].

### Statistical analysis

Patient sociodemographic and disease characteristics were compared between incident minocycline and/or doxycycline users and those never exposed using data from their initial NDB observation. For the continuous study variables, means and standard deviations were calculated and then analyzed with unpaired *t *tests. For dichotomous data, a chi-square test was performed. A two-tailed *P *value of 0.05 was considered significant.

A time-to-event analysis was conducted with initiation of either minocycline or doxycycline as the event of interest. For this analysis, we eliminated all starters who had taken either drug prior to NDB enrollment. Cox proportional hazards regression models were used to test a number of potential covariates, including demographics, disease characteristics, and drug-use history. A final model was developed using all significant predictors with age and gender included for control purposes. A global test for violation of the proportional hazards assumption confirmed that the assumption was not violated.

All patients with minocycline and/or doxycycline exposure, regardless of initiation prior to or during NDB enrollment, were evaluated for self-reported side effects. The prevalence of specific side effects was determined and categorized by organ system and severity. The frequencies with which side effects led to drug discontinuation, dose adjustment, the addition of medications, a visit to a physician, missed work, and hospitalization were also calculated.

The study was carried out in compliance with the Helsinki Declaration, and was approved by the Institutional Review Board of the St Francis Regional Medical Center, Wichita, KS, USA. All patients signed an informed consent.

## Results

**Figure 1:**
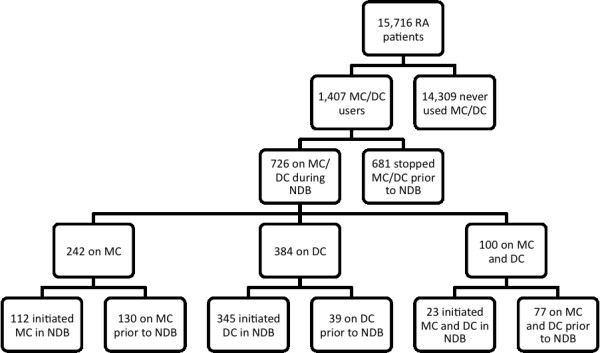
**Rheumatoid arthritis patients with minocycline and doxycycline exposure**. Flow diagram of National Data Bank for Rheumatic Diseases (NDB) rheumatoid arthritis (RA) patients with regards to minocycline (MC) and doxycycline (DC) exposure.

A total of 15,716 RA patients were evaluated (Figure 1). Of these patients, 1,407 (9.0%) received minocycline or doxycycline at some time during their disease course, with 726 (4.6%) receiving either drug during direct NDB observation. There were 480 (3.1%) incident users (minocycline *n *= 112; doxycycline *n *= 345; both drugs *n *= 23). Patients were seen by a total of 1,067 rheumatologists, of which 196 (18.4%) prescribed either treatment (minocycline *n *= 79, doxycycline *n *= 162, both drugs *n *= 45). The median number of patients per doctor on either treatment was one patient (interquartile range one to two patients). The upper decile of rheumatologists had five or more patients on these agents. The median daily dose for both minocycline and doxycycline was 200 mg (interquartile range 100 to 200 mg). The median duration of therapy for minocycline was 6 months (interquartile range 2 to 15 months) and for doxycycline was 3 months (interquartile range 1 to 6 months).

Table 1 summarizes the sociodemographic information, RA characteristics, and concomitant medications of patients initiating minocycline and/or doxycycline therapy and those patients never exposed. Minocycline and doxycycline users were slightly younger (58.35 years vs. 59.76 years, *P *= 0.021) and more commonly non-Hispanic Caucasian (93.68% vs. 89.72%, *P *= 0.005) than nonusers. They had longer duration of RA (14.77 years vs. 13.70 years, *P *= 0.036), an increased number of lifetime DMARDs (2.90 vs. 2.13, *P *< 0.001) and lifetime combined DMARD and biologic agents (3.30 vs. 2.52, *P *< 0.001), and were more frequently on prednisone (40.08% vs. 35.34%, *P *= 0.033). Minocycline and doxycycline users had a different insurance profile (*P *= 0.044) and their mean physical component summary score was lower (indicating worse physical functioning; 35.03 vs. 36.39, *P *= 0.008) compared with those without tetracycline exposure. There were no other study variables that reached statistical significance. After excluding patients who initiated both minocycline and doxycycline during NDB observation (*n *= 23), there were no significant differences in study variables between incident minocycline and doxycycline users (data not shown). In multivariable Cox regression, initiation of either treatment was associated with an increase in comorbidities, previous number of nonbiologic DMARDs, calendar year, and disease activity (as measured by the Patient Activity Scale), and with a decrease in previous number of biologic DMARDs and current use of methotrexate, leflunomide, or azathioprine (Table 2).

**Table 1:** Patient characteristics of minocycline and doxycycline users and nonusers

	Initiated MC/DC (*n *= 480)	Never used MC/DC (*n *= 14,309)	*P *value
Age (years)	58.35 (11.60)	59.76 (13.28)	**0.021**
Male sex (%)	20.83	22.23	0.469
Rheumatoid arthritis duration (years)	14.77 (10.92)	13.70 (11.05)	**0.036**
Education (years) (0 to 17)	13.70 (2.32)	13.52 (2.33)	0.106
Caucasian, non-Hispanic (%)	93.68	89.72	**0.005**
Income (US$)	46,698 (28,088)	46,309 (29,352)	0.775
Semi-annual costs (US$)	6,007 (10,635)	5,685 (7,835)	0.406
Semi-annual drug costs (US$)	4,073 (8,522)	3,826 (6,528)	0.098
Insurance (%)			**0.044**
Private	29.17	27.26	
Health maintenance organization	11.25	8.64	
Medicare	41.67	47.10	
Preferred provider organization	10.00	9.27	
Medicaid	4.79	5.77	
No insurance	3.13	1.95	
Lifetime DMARDs	2.90 (1.83)	2.13 (1.54)	**< 0.001**
Lifetime DMARDs and biologics	3.30 (2.10)	2.52 (1.81)	**< 0.001**
Prednisone (%)	40.08	35.34	**0.033**
Methotrexate (%)	49.48	50.48	0.667
Comorbidity index (0 to 9)	1.68 (1.46)	1.59 (1.48)	0.193
Health Assessment Questionnaire (0 to 3)	1.06 (0.71)	1.04 (0.73)	0.474
Physical component summary score (0 to 100)	35.03 (11.17)	36.39 (11.09)	**0.008**
Mental component summary score (0 to 100)	50.02 (11.30)	49.11 (11.49)	0.086
Patient global (0 to 10)	3.47 (2.50)	3.50 (2.53)	0.784
Pain (0 to 10)	3.92 (2.82)	3.83 (2.79)	0.506
Patient Activity Scale (0 to 10)	3.64 (2.27)	3.60 (2.24)	0.666

Data presented as mean (standard deviation). DC, doxycycline; DMARD, disease-modifying anti-rheumatic drug; MC, minocycline.

**Table 2:** Multivariable Cox proportional hazards model for time to initiation of minocycline or doxycycline

	Hazard ratio	95% confidence interval	Standard error	*P *value
Male sex	1.139	0.887, 1.464	0.146	0.308
Age	0.990	0.982, 0.998	0.004	0.021
Number of previous nonbiologic DMARDs	1.618	1.532, 1.710	0.045	< 0.001
Methotrexate use	0.728	0.596, 0.890	0.074	0.002
Leflunomide use	0.622	0.470, 0.822	0.089	0.001
Azathioprine use	0.378	0.186, 0.769	0.137	0.007
Patient Activity Score	1.049	1.000, 1.100	0.026	0.051
Comorbidity index	1.139	1.072, 1.210	0.035	< 0.001
Number of previous biologic DMARDs	0.779	0.676, 0.898	0.057	0.001
Calendar year	1.062	1.001, 1.127	0.032	0.046

*n *= 11,987. DMARD, disease-modifying anti-rheumatic drug.

**Figure 2:**
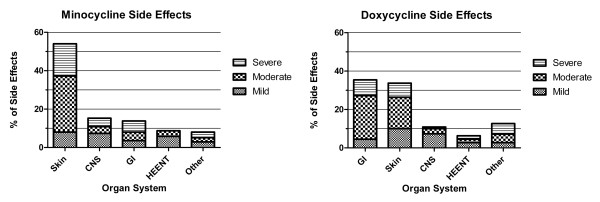
**Minocycline and doxycycline side effects by organ system and severity**. Percentage of **(a) **minocycline and **(b) **doxycycline side effects by organ system and severity. CNS, central nervous system; GI, gastrointestinal; HEENT, Head Eyes Ears Nose Throat.

There were 137 side effects reported by 61 out of 342 (17.8%) minocycline users and 100 side effects reported by 57 out of 484 (11.8%) doxycycline users. The frequency and severity of specific treatment-related side effects with minocycline and doxycycline are summarized in Table 3, while the frequency and severity of these side effects by organ system are shown in Figure 2. Minocycline side effects most commonly involved the skin (54%); the other most common adverse minocycline effects were dizziness (9.5%) and nausea (5.1%). The most common doxycycline side effects were nausea (15.5%), other skin abnormalities (10%), photosensitivity (8.2%), and dizziness (8.2%). The majority of side effects were classified as mild or moderate for minocycline (70.0%) and doxycycline (76.4%).

**Table 3:** Most common rheumatoid arthritis patient-reported side effects to minocycline and doxycycline and their severity

Side effect	Mild	Moderate	Severe	Total (%)
Minocycline				
Skin, other	7	16	11	34 (25)
Photosensitivity	2	9	4	15 (11)
Purpura	2	10	1	13 (10)
Dizziness	8	2	3	13 (10)
Rash	0	3	5	8 (6)
Nausea	1	2	4	7 (5)
Headache	1	1	3	5 (4)
Tinnitus	3	1	0	4 (3)
Diarrhea	2	1	1	4 (3)
Itching	0	2	1	3 (2)
Gastrointestinal, other	0	2	1	3 (2)
Abdominal pain	1	1	1	3 (2)
Other	11	8	6	25 (18)
Total (%)	38 (28)	58 (42)	41 (30)	137 (100)
Doxycycline				
Nausea	3	10	4	17 (16)
Skin, other	7	2	2	11 (10)
Photosensitivity	2	4	3	9 (8)
Dizziness	6	2	1	9 (8)
Rash	0	5	3	8 (7)
Abdominal pain	0	5	2	7 (6)
Purpura	2	4	0	6 (6)
Diarrhea	2	2	2	6 (6)
Itching	0	3	0	3 (3)
Tinnitus	2	1	0	3 (3)
Heartburn	0	2	1	3 (3)
Infections	0	1	2	3 (3)
Other	7	12	6	25 (23)
Total (%)	31 (28)	53 (48)	26 (24)	110 (100)

Table 4 summarizes the patient-reported consequences of medication side effects for all patients exposed to minocycline and/or doxycycline. For minocycline, adverse drug effects led to a doctor visit in 48.2%, discontinuation in 38.2%, the use of additional medication in 32%, a dose change in 31.6%, missed work in 1.1%, and hospitalizations in 1.0% of the patients who reported a medication side effect. For doxycycline, side effects caused a doctor visit in 59.5%, the use of additional medication in 52.8%, discontinuation in 42.7%, a dose change in 16.7%, missed work 6.8%, and hospitalizations in 1.3%.

**Table 4:** Consequence of patient-reported side effects for minocycline and doxycycline

	Minocycline (*n *= 102)	Doxycycline (*n *= 82)
Discontinued (%)	38.2	42.7
Dose change (%)	31.6	16.7
Additional medication (%)	32.0	52.8
Doctor visit (%)	48.2	59.5
Missed work (%)	1.1	6.8
Hospitalized (%)	1.0	1.3

## Discussion

Recent investigations have provided evidence that tetracyclines are moderately effective drugs in the treatment of early RA [7-12,15,16]. Their efficacy appears to be much less robust in long-standing disease. Despite this, the mean duration of RA for NDB participants initiating minocycline or doxycycline was 14.8 years; this is longer than previous studies by Kloppenburg and colleagues (12 ± 10 years) and Tilley and colleagues (8.4 ± 8.6 years), in which long-standing disease showed only modest improvement with minocycline treatment [8,9]. Additionally, minocycline and doxycycline users in the current study probably had refractory RA, as indicated by more frequent prednisone use and increased lifetime exposure to DMARDs and biologic agents. This was supported by the Cox regression data, which showed that patients initiating minocycline and/or doxycycline had increased disease activity and greater cumulative exposure to nonbiologic DMARDs. These findings are similar to a small retrospective analysis of minocycline at a community hospital, in which the mean duration of RA was 18 years and patients had failed treatment with two to eight other DMARDs [26]. Although statistically significant, it is unlikely that small differences in age, race, physical component summary score, and insurance type are clinically relevant. These findings suggest that providers have not embraced tetracyclines as primary treatment options for RA and reserve their use primarily as salvage therapy in patients with long-standing disease that have failed other agents. These results stand in contrast to double-blind, placebo-controlled trials in early, DMARD-naïve RA patients in which minocycline resulted in significant short-term disease improvement [10] and long-term remission [11], and proved more efficacious than hydroxychloroqine [12]. Likewise, doxycycline in combination with methotrexate has proven more efficacious than methotrexate monotherapy [15]. Moreover, results of a meta-analysis of tetracyclines found meaningful effects on tender and swollen joint counts, erythrocyte sedimentation rate, and patient-reported pain [16], comparable with hydroxychloroquine [27], sulfasalazine [28], and gold [29].

Contemporary studies of minocycline and doxycycline in RA coincided with the emergence of biologics. Rheumatologists now have an expanding arsenal of these potent agents available, in addition to conventional DMARDs. Antibiotics are generally considered late in the disease process after other standard treatments have failed. While we do not argue that minocycline and doxycycline should be used as first-line agents, they should be considered with other second-line DMARDs as options for combination therapy with methotrexate and for patients who are reticent to try conventional therapies or cannot afford them. Additionally, recent research into the possible role of oral filamentous bacteria in the pathogenesis of RA may renew interest in the therapeutic role of antibiotics [30-34].

Despite concerns about drug toxicity, the current study found that minocycline and doxycycline are generally well tolerated. Side effects were reported by only 17.8% of minocycline users and 11.8% of doxycycline users, most of which were of moderate severity. Less than one-half of these patients discontinued minocycline or doxycycline because of side effects, suggesting that they are at least as well tolerated as other second-line RA medications, for which drug toxicity causes discontinuation in 15% of all patients [35]. For minocycline, cutaneous side effects were the most common, accounting for over one-half of all patient reports. This is consistent with previous research, in which hyperpigmentation has been reported in 40% of chronic minocycline users [36,37]. Since we did not have dedicated coding for hyperpigmentation, we speculate that this side effect was variously reported as skin, other, photosensitivity, purpura, and rash. Dizziness was reported less frequently than in previous reports, accounting for only about 10% of all reported side effects [8,9,38]. Doxycycline side effects were also similar to other studies, with gastrointestinal and skin manifestations being most common [38]. Interestingly, dizziness was nearly as common with doxycycline use (8.2%) as with minocycline (9.5%).

Nearly one in five rheumatologists had prescribed minocycline or doxycycline, and about 10% of patients had used them at some point in their disease course. However, most doctors had only prescribed these treatments for one or two patients. Although we do not have data to explain this pattern, it is possible that tetracyclines are used to treat a niche population of RA patients, such as those who are reticent to try more conventional DMARDs for fear of potential toxicities or those who may have requested from their physician an antibiotic-based treatment. The median duration of minocycline (6 months) and doxycycline (3 months) treatment was shorter than for other second-line DMARDs [39]. This is not surprising given that our cohort had long-standing, refractory disease, thus limiting the efficacy of any treatment option. Additionally, short-term use of minocycline and doxycycline for treatment of infectious processes may have skewed our results. For those who initiated minocycline or doxycycline during NDB observation, 28% were stopped following the first month of treatment, with side effects accounting for 17% of these discontinuations. Since it is unlikely that minocycline and doxycycline would be stopped so quickly if initiated as DMARDs, some patients probably received these drugs for short-term antibiotic use. When patients with courses of minocycline and doxycycline equal to or less than 30 days were eliminated via a sensitivity analysis (*n *= 133), however, the sociodemographic and disease characteristic results were not meaningfully changed (data not shown). Excluding patients with 1 month or less of treatment had only modest effects on the median duration of minocycline (7 months) and doxycycline (6 months) therapy.

The present study had several limitations. In the analysis of socioeconomic and disease characteristics, minocycline and doxycycline users were combined to improve power. These two groups possibly have distinctive qualities, although there were no significant differences when we compared minocycline and doxycycline users with one another (data not shown). The current analysis also did not have access to laboratory markers such as the rheumatoid factor status of patients; seropositivity may be predictive of minocycline and doxycycline efficacy in early RA [10-12,15], thus impacting timing of drug initiation. Finally, our findings may not be generalizable to racial/ethnic minorities as Caucasian patients made up about 90% of our cohort, with African Americans and Hispanic patients accounting for 4.7% and 2.9% of the sample, respectively. Despite these limitations, this is to our knowledge the first study to examine minocycline and doxycycline use in a large number of community-dwelling RA patients. Most clinical trials of minocycline and doxycycline in RA have been fairly small, and systematic reviews of drug toxicities have relied upon case reports and trials with diverse dosing strategies, patient populations, and drug indications. By focusing on community-dwelling patients, we were able to investigate the real-world application of tetracycline therapy in RA.

## Conclusions

In summary, these data provide evidence that rheumatologists have not embraced minocycline and doxycycline as primary treatment options for early, DMARD-naïve RA. Patients initiating minocycline or doxycycline therapy tend to have longer disease duration, more prednisone use, and exposure to a greater number of DMARDs, which suggests long-standing, refractory disease. While minocycline or doxycycline is prescribed by one in five rheumatologists, most use them infrequently in only one to two RA patients. The duration of minocycline and doxycycline therapy was 6 months and 3 months, respectively. Both minocycline and doxycycline are generally well tolerated, with skin abnormalities, nausea, and dizziness being the most common patient-reported side effects.

## Abbreviations

DMARD: disease-modifying anti-rheumatic drug; IL: interleukin; NDB: National Data Bank for Rheumatic Diseases; RA: rheumatoid arthritis; TNF: tumor necrosis factor.

## Competing interests

The authors declare that they have no competing interests.

## Authors' contributions

CJS participated in the design of the study and wrote the initial draft. HS performed statistical analysis. TRM participated in the design of the study and assisted in revising drafts. KM conceived the study, participated in statistical analysis, and assisted in revising drafts. All authors read, contributed to, and approved the final manuscript.
